# Diagnostic Accuracy of a Computed Tomography-Guided Transthoracic Needle Biopsy for Ground-Glass Opacities and Subsolid Pulmonary Nodules

**DOI:** 10.7759/cureus.57414

**Published:** 2024-04-01

**Authors:** Anoop Koratala, Nikitha C Chandra, Prasanth Balasubramanian, Alejandra Yu Lee-Mateus, Alanna Barrios-Ruiz, Ana Garza-Salas, Andrew Bowman, Rolf Grage, Sebastian Fernandez-Bussy, David Abia-Trujillo

**Affiliations:** 1 Pulmonary, Allergy, and Sleep Medicine, Mayo Clinic, Jacksonville, USA; 2 Pulmonary and Critical Care Medicine, Mayo Clinic, Jacksonville, USA; 3 Radiology, Mayo Clinic, Jacksonville, USA

**Keywords:** computed tomography-guided core needle biopsy, diagnostic accuracy, lung cancer, subsolid nodule, ground-glass nodule

## Abstract

Purpose

The increasing use of computed tomography (CT) imaging has led to the detection of more ground-glass nodules (GGNs) and subsolid nodules (SSNs), which may be malignant and require a biopsy for proper diagnosis. Approximately 75% of persistent GGNs can be attributed to adenocarcinoma in situ or minimally invasive adenocarcinoma. A CT-guided biopsy has been proven to be a reliable procedure with high diagnostic performance. However, the diagnostic accuracy and safety of a CT-guided biopsy for GGNs and SSNs with solid components ≤6 mm are still uncertain. The aim of this study is to assess the diagnostic accuracy of a CT-guided core needle biopsy (CNB) for GGN and SSNs with solid components ≤6 mm.

Methods

This is a retrospective study of patients who underwent CT-guided CNB for the evaluation of GGNs and SSNs with solid components ≤6 mm between February 2020 and January 2023. Biopsy findings were compared to the final diagnosis determined by definite histopathologic examination and clinical course.

Results

A total of 22 patients were enrolled, with a median age of 74 years (IQR: 68-81). A total of 22 nodules were assessed, comprising 15 (68.2%) SSNs with a solid component measuring ≤6 mm and seven (31.8%) pure GGNs. The histopathological examination revealed that 12 (54.5%) were diagnosed as malignant, nine (40.9%) as benign, and one (4.5%) as non-diagnostic. The overall diagnostic accuracy and sensitivity for malignancy were 86.36% and 85.7%, respectively.

Conclusion

A CT-guided CNB for GGNs and SSNs with solid components measuring ≤6 mm appears to have a high diagnostic accuracy.

## Introduction

A pulmonary nodule is defined as a small (≤3 cm in diameter) and focal radiographic opacity that is surrounded by lung parenchyma [1]. Based on appearance, pulmonary nodules are categorized as solid and subsolid. Subsolid pulmonary nodules (SSNs) include pure ground-glass nodules (GGNs) and part-solid nodules. A GGN is defined as a hazy attenuation of a lung with visible bronchial and vascular structures [2]. Part-solid nodules are the nodules with both ground-glass and solid components [2].

SSNs, including those composed solely of GGNs, are being more commonly recognized in clinical practice due to the increased utilization and enhanced resolution of computed tomography (CT) imaging. The incidence of a subsolid nodule is reported to be 4.2% in baseline screening for lung cancer and 0.7% in annual repeat screening [3]. Approximately 80% of persistent GGNs can be attributed to adenocarcinoma in situ or minimally invasive adenocarcinoma [4]. Adenocarcinoma in situ refers to adenocarcinoma consisting entirely of the lepidic/bronchioloalveolar pattern, while minimally invasive adenocarcinoma refers to an invasive adenocarcinoma with a predominantly lepidic growth pattern and a maximum invasive size of 3 cm or less [5]. However, it should be noted that the differential diagnosis of GGN is not limited to malignancy alone, as it may also comprise premalignant lesions such as atypical adenomatous hyperplasia and non-neoplastic conditions such as focal fibrosis and inflammation [6]. Biopsy and tissue sample acquisition are imperative to rule out malignancy and to determine the nature of the lesion.

A CT-guided core needle biopsy (CNB) is a minimally invasive procedure for the diagnosis of thoracic lesions. It is an essential tool for confirming the neoplastic nature of the lesion and characterizing its genotypic and molecular profile. A CT-guided biopsy has been proven a reliable procedure, with reported achieved diagnostic accuracy rates of 93% for SSNs with solid components of any size [7]. However, the diagnostic accuracy and safety of a CT-guided biopsy for GGNs and SSNs with solid components ≤6 mm are still uncertain when compared to solid nodules.

The aim of this study was to assess the diagnostic accuracy of a CT-guided CNB for GGNs and SSNs with solid components ≤6 mm.

## Materials and methods

This is a retrospective analysis conducted at Mayo Clinic, Florida, United States, on patients who underwent a CT-guided CNB for the evaluation of pulmonary nodules between February 2020 and January 2023. The inclusion criteria were the presence of GGNs or SSNs with a solid component measuring ≤6 mm. We recorded patients' baseline characteristics, procedure details, assessment of the nodules, and complications encountered during the biopsy. The data were encrypted and stored in a secure database. The study received approval from the Institutional Review Board (IRB # 22-010644). The primary outcome was to determine the efficacy and diagnostic performance of a CT-guided CNB for diagnosing GGNs and SSNs. The secondary goals were to evaluate the safety of this procedure, as determined by the incidence of complications that occurred following the biopsy.

CT-guided CNB

Siemens Healthineers' SOMATOM scanners (Siemens Healthineers; Siemens Medical Solutions, Malvern, PA) were used for the CT-guided CNB procedures. Prior to the biopsy, a thorough analysis of pre-procedure CT scans determined the optimal sampling route considering nodule location, patient positioning, and needle entry site and angle to avoid fissured areas, bullae, and substantial aerated pulmonary parenchyma. Anesthesia was administered locally with 1% lidocaine solution, and needle sizes for biopsy ranged from 18G to 20G. All patients underwent a tissue core biopsy with a coaxial technique, followed by rapid on-site evaluation (ROSE) for sample adequacy and preliminary diagnosis. Additional passes were made if necessary. Post-procedure, chest radiography was performed within two to four hours to assess pneumothorax. Patients followed the institution's recovery protocol before discharge.

Complications

Adverse events associated with the intervention were documented within a seven-day post-procedural period. The two most common complications were pneumothorax and hemoptysis. Pneumothorax was considered an adverse effect if it led to hospitalization for observation or required chest tube placement, as defined by established criteria [8]. Hemoptysis was categorized using the Nashville Bleeding Scale, where bleeding was labeled as mild, moderate, or severe according to escalating interventions to control bleeding, from suctioning to advanced ventilatory support, transfusion, or resuscitation, and procedure abortion [9].

Histopathological diagnosis and follow-up

All procedures in this study included ROSE to assess sample adequacy, with a cytotechnologist present for immediate pathological interpretation. Subsequently, biopsy findings were cross-referenced with the conclusive diagnosis through definitive histopathologic examination, immunohistochemical staining, and progression of the clinical course. In instances where an initial definite diagnosis could not be established, secondary procedures such as re-intervention, surgical resection, alterations in CT, or if there was evidence of a malignant clinical progression provided the final diagnosis.

Statistical analysis

Clinicodemographic information was presented using medians and interquartile ranges for quantitative data values, while categorical variables were expressed as counts and percentages. Descriptive analysis of the data was conducted using Microsoft Excel and SPSS Statistics (version 28.0). Diagnostic accuracy was evaluated as follows: biopsy samples were categorized as true positives (TP) if they resulted in a definitive malignant diagnosis or suspicion of malignancy confirmed through a subsequent procedure or demonstrated malignant progression - growth in size or number - at six months follow-up or later. True negatives (TN) were assigned to cases with a conclusive benign diagnosis or those negative for malignancy, subsequently confirmed as benign through a secondary procedure or exhibiting benign clinical progression, including lesion resolution, size reduction, or stability, at six months follow-up or later. False positives (FP) were characterized by initial malignant diagnosis, but subsequent investigation or clinical course revealed benign disease at six months follow-up or later. False negatives (FN) comprised cases with a benign pathology or negative for malignancy that, following a secondary procedure or clinical progression, were confirmed as malignant at six months follow-up or later. The results were tabulated in a 2 x 2 table for sensitivity, specificity, positive predictive value, and negative predictive value estimations.

## Results

In this study, a total of 22 patients were enrolled, with a median age of 74 years (IQR, 68-81) and a median BMI of 25.5 kg/m^2^ (IQR: 21.2-27.8). Of the participants, 16 (72.7%) were female, and 10 (45.5%) had a personal history of cancer, but none were lung cancer. General demographic information, along with patients’ smoking history, is presented in Table 1.

**Table 1:** Demographic and clinical characteristics. Quantitative data are presented as median and interquartile range.

Demographic and Clinical Characteristics	CT-Guided CNB (N=22)
Age	74 (68, 81)
BMI	25.5 (21.2, 27.8)
Gender	
Female	16 (72.27%)
Male	6 (27.3%)
Personal History of Cancer	
No	12 (54.5%)
Yes	10 (45.5%)
Personal History of Lung Cancer	
No	22 (100%)
Yes	0 (0%)
Smoking History	
Former	18 (81.8%)
Never	3 (13.6%)
Current	1 (4.5%)
Pack-years	26.3 (18,38) ​​​​​

**Figure 1:**
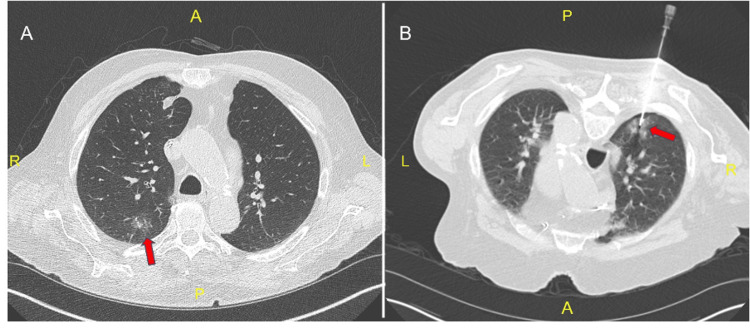
A) Chest computed tomography axial view showing a 20 x 14 mm sub-solid nodule with solid components measuring 4 mm in the right upper lobe. B) Computed tomography image obtained during a CT-guided biopsy, showing a biopsy needle targeting a subsolid nodule in the right upper lobe.

**Figure 2:**
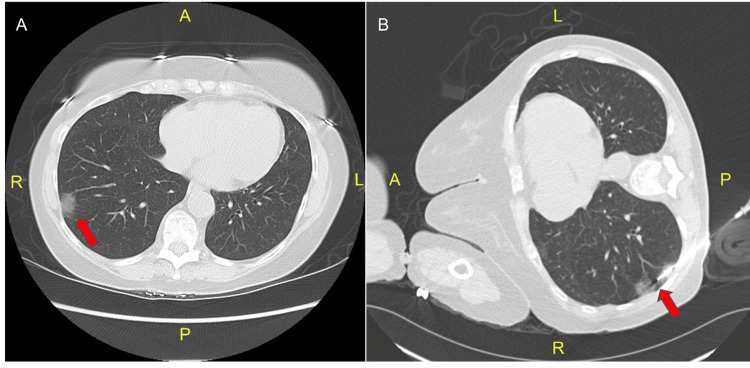
A) Chest computed tomography axial view showing a 23 x 14 mm ground-glass nodule in the right lower lobe. B) Computed tomography image obtained during a CT-guided biopsy, showing a biopsy needle targeting the ground-glass nodule in the right lower lobe.

A total of 22 nodules were assessed, comprising 15 (68.2%) SSNs, with a solid component measuring ≤6 mm (Figure 1), and seven (31.8%) pure GGNs (Figure 2, Table 2). The majority of target nodules (9, 40.9%) were located in the left upper lobe. The nodules had a median maximum cross-sectional diameter of 1.4 cm (IQR: 1.1-1.6), and a minimum cross-sectional diameter of 0.9 cm (IQR: 0.7-1.1). SSNs had a median maximum diameter of solid components of 0.6 cm (IQR: 0.5-0.6) and a minimum diameter of 0.35 cm (IQR: 0.3-0.43). The median distance from the skin to the nodule was 6.53 cm (IQR: 5.94-7.6), while the median distance from the pleura was 1.04 cm (IQR: 0.4-1.8).

**Table 2:** Ground-glass opacities and subsolid nodules characteristics. Quantitative data are presented as median and interquartile range.

Nodule Characteristics	CT-Guided CNB (N=22)
Nodule Location	
LUL	9 (40.9%)
RUL	7 (31.8%)
RLL	5 (22.7%)
RML	1 (4.5%)
LLL	0 (0%)
Nodule Type	
Part solid	15 (68.2%)
Ground-glass	7 (31.8%)
Nodule size maximum (cm)	1.4 (1.1, 1.6)
Nodule size minimum (cm)	0.9 (0.7, 1.1)
Solid component size maximum (cm)	0.6 (0.5, 0.3)
Solid component size minimum (cm)	0.35 (0.3, 0.43)
Distance from skin (cm)	6.53 (5.94, 7.6)
Distance to pleura (cm)	1.04 (0.4, 1.8)

The median duration of the procedure was 25.5 minutes (IQR: 20-36). Among the complications encountered, pneumothorax was the most common (seen in nine (40.9%) patients), followed by bleeding (seen in two (9.1%) patients). A total of 5 (22.7%) patients required admission for pneumothorax, of which two required chest tubes (Table 3).

**Table 3:** CT-guided core needle biopsy procedure details. Quantitative data are presented as median and interquartile range.

CT-Guided CNB Procedure Details	CT-Guided CNB (N=22)	
Biopsy Needle Size		
18G	19 (86.4%)	
20G	3 (13.6%)	
Number of Needle Passes		
1	3 (15.8%)	
2	10 (52.6%)	
3	3 (15.8%)	
4	2 (10.5%)	
5	1 (4.5%)	
Duration of the Procedure in Minutes	26.5 (20, 36)	
Complications		
Pneumothorax		9 (40.9%)
Hemorrhage		2 (9.1%)
Others		0 (0%)
Chest Tube Placement		
No	20 (90.9%)	
Yes	2 (9.1%)	
Hospital Admission		
No	17 (77.3%)	
Yes	5 (22.7%)	
Length of stay in hospital, days	0 (0,0)	

The histopathological examination of 22 biopsy samples revealed that 12 (54.5%) were diagnosed as malignant, nine (40.9%) as benign, and one (4.5%) as non-diagnostic. For detecting malignancy, overall sensitivity, specificity, positive predictive value (PPV), negative predictive value (NPV), and diagnostic accuracy were found to be 85.7%, 100%, 100%, 77.7%, and 86.36%, respectively (Table 4).

**Table 4:** Diagnostic performance of a CT-guided core needle biopsy for ground-glass opacity and subsolid nodules.

Pathology Report	
Malignant	12 (54.5%)
Benign	9 (40.9%)
Non-diagnostic	1 (4.5%)
Malignancy diagnosis	
True Positive	12
False Positive	0
True Negative	7
False Negative	2
Diagnostic Accuracy	
Sensitivity	85.7%
Specificity	100%
Positive Predictive Value	100%
Negative Predictive Value	77.7%

## Discussion

The diagnostic performance of a CT-guided CNB was assessed in 22 SSNs, consisting of seven GGNs and 15 part-solid nodules with a solid component measuring ≤6 mm, at our tertiary care referral center. Although a CT-guided CNB has been established as a reliable and highly accurate diagnostic method for lung nodules, the diagnostic sensitivity for SSNs was found to be relatively low when compared to solid nodules [10]. This may be attributed to the lower cellular density of SSNs compared to solid nodules [10]. Furthermore, fewer studies have been conducted on nodules ≤1 cm [11,12], and no clear data are available for diagnostic accuracy of part-solid nodules with solid components ≤6 mm using a CT-guided CNB.

Recent advancements in low-dose CT screening protocols have led to the detection of an increasing number of SSNs and smaller nodules, resulting in the diagnosis of cancer in smaller nodules [13,14]. It has been shown that the size of the pulmonary nodule is a determining factor for the diagnostic accuracy of a CT-guided CNB [15].

The present study revealed an overall sensitivity of 85.7%, specificity of 100%, PPV of 100%, NPV of 77.7%, and a diagnostic yield of 86.36%. These findings were comparable to earlier studies that used a CT-guided CNB as the diagnostic tool [7,15,16]. However, in our study, the nodules were smaller, and we were able to preserve a high diagnostic accuracy despite specifically targeting pure GGNs and SSNs with solid components ≤6 mm. Previous research by Shimizu et al. indicated that a CT-guided aspiration biopsy had lower diagnostic yields for GGN-dominant lesions when compared to solid lesions [17]. Additionally, Hur et al. reported that diagnostic accuracy was notably decreased by the presence of a larger GG component [18]. The observed differences in accuracy could potentially be attributed to the lower cellularity of aspirates obtained from part-solid lesions [19]. Our study found a higher diagnostic yield despite we performed biopsies on GG-predominant lesions. It is noteworthy that our study employed core CNB, which is capable of obtaining adequate core specimens, while previous studies that reported lower diagnostic yields for lesions with a predominant GGN component utilized a fine needle aspiration biopsy [17,18]. The use of CNB may have contributed to the higher diagnostic accuracy observed in our study by enabling the acquisition of larger and more representative tissue samples.

The diagnostic accuracy of CNB for lesions with varying GG component proportions remains a subject of debate in the literature. For instance, Yamagami et al. reported that the diagnostic accuracy of CNB differed significantly with the proportion of GG component, even though CNB was used [20]. In contrast, Kim et al. reported conflicting results, indicating that the diagnostic accuracy of CNB was not influenced by the proportion of GG components. Both studies analyzed the differences in diagnostic accuracy according to the GG component proportion of part-solid lesions [16]. The conflicting results of these studies suggest that further research is needed to clarify the diagnostic accuracy of CNB for lesions with varying GG component proportions.

In the present study, the most frequently encountered complication was pneumothorax, which occurred in 40.9% of cases. The incidence of pneumothorax reported in previous studies varied considerably, ranging from 15% to 50% [21,22], regardless of the nature of the nodule. Among the cases of pneumothorax observed in our study, only two required chest tube insertion, while the remaining cases resolved spontaneously. Hemorrhage was the second most common complication, occurring in two cases. The severity of the hemorrhage was low and did not need further interventions.

The study presented several limitations that should be taken into consideration when interpreting the results. Firstly, it was a retrospective study, which might have introduced selection bias. To minimize this bias, future studies should consider using a prospective design. Secondly, the study was conducted at a single center, which may limit the generalizability of our findings. It is possible that the patient population at this center may differ from other centers, which could affect the diagnostic yield and complication rates. Therefore, future studies should involve multiple centers to increase the external validity of the results or compare them with other alternative sampling strategies such as robotic-assisted bronchoscopy. Thirdly, the study had a relatively small sample size as we focused only on GGNs and had a strict cut-off for solid components, which may limit the statistical power of the analysis. As a result, the study may not have been able to detect small but clinically significant differences in diagnostic yield or complication rates. To address this limitation, future studies should aim to enroll larger sample sizes.

## Conclusions

Our study assessing the diagnostic performance of CT CNB for small SSNs with solid components ≤6 mm demonstrated a notable overall sensitivity of 85.7% and diagnostic yield of 86.36%. Despite the inherent challenges posed by smaller nodules, our results were comparable to prior studies using CNB for larger lesions. Particularly, our focus on pure GGNs and SSNs with a small solid component did not compromise diagnostic accuracy. Further studies comparing alternative approaches are needed.
